# Integrating climate, biodiversity, and sustainable land-use strategies: innovations from China

**DOI:** 10.1093/nsr/nwaa139

**Published:** 2020-06-23

**Authors:** Guido Schmidt-Traub, Harvey Locke, Jixi Gao, Zhiyun Ouyang, Justin Adams, Lin Li, Enric Sala, M Rebecca Shaw, Sebastian Troëng, Jing Xu, Chunquan Zhu, Changxin Zou, Tianxiao Ma, Fuwen Wei

**Affiliations:** Sustainable Development Solutions Network, France; Beyond the Aichi Targets Task Force IUCN World Commission on Protected Areas, Switzerland; Yellowstone to Yukon Conservation Initiative, Canada; Center for Satellite Applications on Ecology and Environment, Ministry of Ecology and Environment, China; Research Center for Eco-Environmental Sciences, Chinese Academy of Sciences, China; Tropical Forest Alliance, World Economic Forum, Switzerland; Worldwide Fund for Nature, Canada; National Geographic Society, USA; Worldwide Fund for Nature, USA; Conservation International, Colombia; Chinese Research Academy of Environmental Sciences, China; Nature Initiatives and Tropical Forest Alliance, World Economic Forum, China; Nanjing Institute of Environmental Sciences, Ministry of Ecology and Environment, China; Institute of Zoology, Chinese Academy of Sciences, China; Institute of Zoology, Chinese Academy of Sciences, China

To reverse the accelerating degradation of biodiversity and ecosystem services (‘nature’) and climate [1,2], the Convention on Biological Diversity (CBD) and the UN Framework Convention on Climate Change (UNFCCC) will hold major meetings in 2021. We argue that, as a critical priority, countries need to design and implement integrated national strategies to achieve the goals of the three Rio Conventions (including the UN Convention to Combat Desertification—UNCCD) using spatially explicit analyses and policies. This integration can maximize co-benefits and help manage trade-offs to meet the Sustainable Development Goals (SDGs). Nature-based solutions (NBS) provide an important framework for such integration.

An advantage of our proposal is that it requires no negotiations among governments, since available convention instruments can achieve this integration. In particular, long-term low-emission development strategies (LT-LEDS) under the Paris Agreement could play a critical role by requiring a spatially explicit approach to NBS and other land uses. Moreover, China has recently introduced major domestic policy innovations that hold important lessons for designing and implementing spatially explicit policy frameworks to simultaneously pursue the objectives of Rio Conventions. We argue that countries can learn from experiences in China and elsewhere to integrate nature, climate and sustainable management of land and ocean, including targeted conservation and restoration of nature, into their LT-LEDS. We also highlight key design and implementation challenges.

## JOINT IMPLEMENTATION OF THE CONVENTIONS

The three Rio Conventions pursue closely related objectives that integrate across climate, nature and land-use management. These objectives exhibit synergies and some trade-offs. However, national strategies and international policy processes under each convention tend to be siloed and lack the ambition and resources to meet the objectives of the conventions (Supplementary Information—SI).

In 2019, political momentum developed to overcome this artificial separation drawing also on the SDGs, which place the objectives of the Rio Conventions into a broader context. For example, a Canada-hosted Nature Champions Summits called for ‘uniting nature conservation objectives with addressing climate change and developing NBS that are effective for both’. In the run-up to the UN Secretary-General's Climate Summit in September 2015, China and New Zealand led a coalition of countries that adopted the NBS Manifesto to promote NBS as part of the UN climate agenda with backing from the presidents of China and France and other heads of state (SI).

## NBS AND SPATIAL PLANNING

NBS are critical to achieve the objectives of all Rio Conventions. Terrestrial NBS represent about one-third of the potential to reduce net greenhouse-gas emissions [3] and are central to climate adaptation and sustainable water management. Ocean-based mitigation options can make substantial contributions towards a 1.5°C pathway. Many global and national initiatives promote avoided deforestation, reforestation and other NBS, but most efforts are project-based without adequate integration into national strategies. This may generate leakage and trade-offs with other SDG priorities.

Integrating NBS with spatial planning is novel in the context of the Rio Conventions, but intuitive, since NBS relate to broader questions of land use, land management and competition for scarce land, ocean and water resources. For example, protecting a threatened species may require place-based interventions to protect natural habitat, which might have climate benefits and lower the potential for agriculture. Spatial planning needs to address all policy objectives to tackle trade-offs, particularly with regard to biodiversity objectives, or else it may lead to negative outcomes, as has happened in the past with some land-restoration programs in China [4] and elsewhere. Similarly, experiences with protected areas in China [5] and globally show that spatial approaches to managing nature must take into account the needs of local and other affected populations to ensure acceptability and to meet environmental as well as socio-economic objectives, including reducing the risk of new zoonotic diseases (SI).

Despite the need for spatial-policy tools to operationalize NBS, our own review of Nationally Determined Contributions (NDCs) shows that none of the 195 submitted climate strategies include an actionable map. None details how to manage co-benefits and trade-offs with nature and land-degradation objectives. NDCs cover only parts of emissions from land use, land-use change and forestry, often without quantitative targets or clear accounting [6,7] and inadequate treatment of agriculture [8]. Similarly, current national biodiversity strategies under the CBD do not systematically tackle drivers of nature loss, and only 15% include actionable maps for nature's contribution to people [1], including carbon sequestration [9]. The absence of maps and spatial analyses demonstrates that national strategies under the Rio Conventions do not adequately tackle the complex questions of synergies and trade-offs between competing uses for land and sea.

## CHINA’S ECOLOGICAL CIVILIZATION

With 18% of the global population and only 10% of arable land, China is highly vulnerable to loss of agricultural land and nature's contributions to people. Since the 1980s, the country has undertaken some of the largest land-restoration programs in the world [4]. Yet, nature continued to decline precipitously and, in 1998, China experienced catastrophic flooding of the Yangtze River, which killed >4000 people. The realization that these floods had been exacerbated by deforestation, draining of wetlands and other inappropriate land-management practices spurred the government to consider more ambitious and integrated policies to conserve and restore nature [10]. This led to a growing focus on Ecological Civilization—a broad framework for balancing political, economic, social and environmental objectives, which was first mentioned at the 17th CPC National Congress in 2007 and incorporated into the Chinese constitution in 2018.

China has developed national and provincial spatial-zoning plans that cover and integrate functional zones: critical ecological functions, agricultural production and zones for industrial development and human settlements [11]. To strengthen coherence, these initially disparate spatial-planning frameworks are now being consolidated by the Ministry for Natural Resources under a single, integrated land-use-management plan for China to be incorporated into the 14^th^ Five-Year Plan, which take effect in 2021. This will give China a land-use-management framework that integrates across the three Rio Conventions with important lessons for other countries and global policy processes.

These spatial-planning frameworks include ‘redlines’ that delineate areas for special protection or management. As one example, an agricultural redline identifies a minimum agricultural production space of 120 million hectares that must be maintained. Conversion of agricultural land within the agricultural redline is only possible if new agricultural land is brought under production elsewhere in the country. This approach is similar to spatial-zoning regulations in force in many industrialized countries, but the latter do not tend to apply and enforce minimum land requirements for meeting ecological needs at national and provincial levels.

China's Ecological Conservation Redline (ECRL) [10] was first put forward by scientists in 2000, proposed by the State Council in 2011 and then listed as one of the main tasks of Ecological Civilization in 2013. The government uses four steps to identify high-priority areas for biodiversity, ecosystem services and disaster risk reduction covering a quarter of the territory [10]. First, an initial ECRL is identified combining existing protected areas that are important for biodiversity (roughly 18% of the country) with additional priority areas identified through high-resolution mappings of nature using remote sensing and data from 114 500 ground survey sites combined with ecosystem-services modeling [12]. Second, the ECRL is coordinated and aligned with other land-use-planning frameworks, including for agriculture, industry, mining, urban areas and infrastructure. Third, the ECRL is aligned across provinces and coastal areas to ensure the continuity and effective management of cross-boundary ecosystems. Fourth, these ECRL boundaries are then revised in consultation with local governments to balance ecological needs and local development priorities, often leading to significant adjustments to address local concerns [10]. The ECRL covers some 25% of China's land mass.

ECRL management aims to ensure no change in land cover, no net loss of biodiversity and no degradation of other ecosystem services inside the ECRL. Correspondingly, the management practices range from strictly protected areas with no significant human presence to watershed protection areas that can sustain some agriculture and limited other human activities.

By mapping areas of high significance for nature and comparing them with actual and desired socio-economic land uses, the four steps of ECRL design identify potential land-use conflicts across China. For example, the extension of an industrial park designed to generate local employment and revenues might damage a globally significant wetland. Restrictions on dredging may hamper shipping. Reducing the fragmentation of habitat for critical species may call for the relocation of people, which may be resisted. Since local authorities in China derive a large share of their revenues from selling or leasing land, compensatory revenues may be needed in return for the expansion of ECRL.

To compensate local governments for economic losses sustained by putting land inside the ECRL, China is scaling up ‘ecological compensation’ payments including the transfer of RMB 62.7bn ($9.4bn, 0.08% GDP) from the national budget to some 700 counties in 2017 [13]. The country is experimenting with large-scale market-based mechanisms for payments for ecosystem services, which is already widely practiced for watershed management [14].

Another challenge arises from the complexities of land use in China. While land in China is formally under public ownership, land practices vary widely across the country and evolve over time [15]. Some land may be leased by local governments to private entities, farmers may have de facto control over the land they farm and communal lands can be assigned to large numbers of households. When a decision is taken to include a certain area inside the ECRL, complex negotiations are often needed with local authorities to clarify land use and to encourage adherence to the management of ECRL.

Resolving these land-use conflicts has proven complex and time-consuming. In particular, step four of ECRL design—the consultation with local authorities—places high demands on China's administrative capacities. In some cases, such as the newly created Giant Panda National Park, which consolidates smaller protected areas, the government is resettling populations out of the ECRL. As a result, the initial roll-out of ECRLs has experienced numerous delays and implementation is now proceeding more slowly. Some implementation challenges, particularly relating to formal and informal land-use rights, arise out of ECRL management and will need to be addressed gradually. Post-Covid-19 stimulus investments in infrastructure will similarly need to tackle land-use considerations.

ECRLs are scheduled to be fully implemented for 31 provinces and municipalities (out of 34) by the end of 2020. Some of the most densely populated provinces have high proportions of land integrated into the ECRL, e.g. Beijing (26.1%), and Hebei Province (20.7%). Spatial patterns of priority ecosystem services identified through the scientific surveys also form the basis for urban master planning in many cities, such as Beijing and Guangzhou.

China's ECRL and land-use-management framework constitute a highly ambitious and unique policy framework that generates implementation challenges around the need to bala-nce socio-economic and environmental-policy objectives. In addition to these complexities, we see four policy gaps that are specific to China. First, the ECRL does not yet aim to include carbon storage in biomass and soils, though this would require minor adjustments owing to the spatial overlap between biodiversity, ecosystem services and carbon [12]. Work is underway to close this gap, which will make the ECRL a comprehensive policy framework for NBS—possibly with a link to China's emerging Emissions Trading Scheme for greenhouse-gas emissions. Second, today's ECRL does not fully cover marine biodiversity and ecosystems across the country's territorial waters. A marine-conservation redline is under development to fill this gap. Third, like other countries, China lacks policy tools to quantify and minimize its international spillovers, so an effective ECRL may displace unsustainable demand and production practices outside the country's borders. Fourth, policy frameworks are needed to address nature inside intensive agricultural production landscapes not covered by the ECRL.

## APPLYING LESSONS FROM CHINA IN OTHER COUNTRIES

Other countries have mapped nature and land use (SI) but, for several reasons, these efforts have fallen short of the ambition of China's ECRL. In particular, countries have not translated their spatial analyses into integrated national policy frameworks that can meet the objectives of the three Rio Conventions by maximizing the synergies and addressing the trade-offs.

With suitable modifications, China's spatially explicit land-use policies hold important lessons for other countries to design their policy frameworks. The English term ‘redline’ may not have a clear meaning in other countries and it is associated with racial profiling in the USA. So, the terminology might need to be adjusted when applying lessons from China in other countries.

To this end, countries will need to answer two questions.

The first question is how to map nature and human uses of land and ocean. Many governments have the capacity to develop high-resolution maps and all can benefit from freely available global high-resolution maps for terrestrial biodiversity and carbon, other terrestrial ecosystem services and marine ecosystem services. In many instances, maps have been prepared and published by scientists (SI), but have not been formally validated by governments. Such official validations are critical for maps of biodiversity, carbon and other ecosystem services to be used in conjunction with maps of land rights and use.

Such maps of the biophysical resources then need to be combined with maps for urban master plans, industrial development, concessions for extractive industries and other forms of land and resource use to help guide investment decisions, including under the Belt and Road Initiative (BRI). Overlays may show that areas slated for conservation of high biodiversity and ecosystem-services production might be included in mining concessions or industrial development.

The second, far more challenging question is how to design and implement policies for spatial zoning and land use that can manage competition for land use, which often pits economic interests against one another or against objectives for nature conservation and restoration. Major adaptation will be required from the Chinese experience to develop ecological conservation lines that suit other countries’ governance, culture and history. Whereas most land in China is controlled by the government, other countries have extensive private land holdings and other land rights, including rights held by indigenous peoples. Many already operate zoning laws and restrictions to govern construction and other land uses on private land, but few do so with a view towards meeting national objectives for nature conservation. An interesting example for meeting national-scale conservation objectives on private land is Brazil's forest code, which, as long as it was enforced, applied a biodiversity set-aside to large private land holdings. The forest code points to the importance of effective monitoring and enforcement, including through transparent land cadastres and remote sensing of deforestation led by Brazil's National Space Agency (INPE). Weak administrative capacity is another critical challenge in many countries, particularly where competencies for land-use management have been devolved to lower government levels that tend to have less administrative capacity.

Any spatial-planning process will require stakeholder consultation to be widely embraced by government, civil society, indigenous peoples and the private sector. China has conducted extensive consultations and negotiations between national and local authorities to identify and address conflicts between economic, environmental and social objectives [10]. These have encountered numerous challenges, as noted above. Other countries will need to build on their own efforts to promote spatial planning in ways that are appropriate to their circumstances. They can learn from China's design and implementation of its ECRL as part of a comprehensive land-use-planning framework.

## SPATIAL POLICIES IN LONG-TERM CLIMATE STRATEGIES

To integrate implementation of the Rio Conventions, governments can build on experiences in China and other countries to design and implement spatially explicit long-term LT-LEDS, as called for in Article 4.19 of the Paris Agreement. This is an interim step towards fully integrated short-term NDCs that are more operational, but also more difficult to change.

Including spatial policies for nature in LT-LEDS would help address four challenges with meeting the objectives of the three Rio Conventions (SI). It would, first, integrate NBS with the drivers of land-use change and nature loss and, second, raise the visibility of and political attention to nature and sustainable land and ocean management in each country. Third, it would unlock national and international climate finance for nature conservation and restoration projects. And, finally, the integration will strengthen standards for country reporting and transparency on nature and land management.

## GREENING THE BRI AND INTERNATIONAL SUPPLY CHAINS

China is the initiator of the BRI—the largest infrastructure investment program in recent history, covering more than 120 countries. If well designed and executed, BRI investments can support sustainable development in participating countries, as emphasized by President Xi Jinping (SI), but otherwise they may undermine the objectives of the Rio Conventions [16]. Applying lessons from China's domestic spatial policies in BRI countries could provide a framework towards greening the BRI by enabling governments to better site infrastructure investments and manage trade-offs between economic, social and environmental objectives. To ensure coherence with the multilateral environmental agreements and avoid duplication, national efforts to green the BRI should be framed as national strategies under the three Rio Conventions, as outlined above. China can share its domestic learning with BRI partner countries and support capacity development, including for sustainable investment guidelines under the BRI.

Many countries generate international spillovers on other countries through their import of agricultural, forest and other commodities. International initiatives, such as the Tropical Forest Alliance, aim to reduce the environmental impact of individual supply chains, including for oil palm, cattle, soy, pulp and paper or cocoa. Such industry-specific initiatives are critical, but they need to be complemented with domestic spatial-policy frameworks in production countries. This in turn would reduce the ecological footprint of supply chains into China and other importing countries.

## NEXT STEPS

China will host the 2021 CBD COP15 and the CBD Secretariat has announced that the theme will be Ecological Civilization. In addition to crafting an ambitious 2030 goal and a long-term vision, the CBD parties must overcome a persistent weakness in all three Rio Conventions by advancing the implementation of international commitments. As we argue, this requires the inclusion of nature objectives into climate strategies, including through maps and land-use-planning frameworks. There are several opportunities for building momentum.

Based on its domestic leadership, China can promote this integration by announcing the inclusion of its ECRL and land-use-planning frameworks into its LT-LEDS and the submission of this document under the CBD and UNCCD as well. Encouraging BRI partners and key commodity supply countries to apply similar land-use-planning frameworks under the Rio Conventions could make major contributions towards greening the BRI.

France, as host of the Paris Agreement and co-chair (with Costa Rica) of the High-Ambition Coalition for Nature and People that supports a CBD target of at least 30% of the planet protected by 2030, has been promoting the integration of the objectives of the three Rio Conventions in the context of the G7, the G20 and bilaterally with China. The May 2020 EU Biodiversity Strategy contains important measures that support such integration. The September 2021 IUCN World Conservation Congress in Marseille provides an important stage for promoting integrated approaches. The forthcoming China-EU summit can raise ambition and promote the systematic integration of the Rio Conventions.

Many countries, including most industrialized economies, would have to strengthen their domestic policies substantially to match China's level of ambition and integration in pursuing the objectives relating to land-use and ecosystem services under the Rio Conventions. For this reason, they could focus on committing to maps outlining their land-use objectives in the LT-LEDS. Ahead of the 2023 UNFCCC stocktake these maps and policy frameworks for land-use planning could then be integrated into the NDCs.

The UK, working with co-host Italy, have indicated that nature will be a major focus of the Glasgow COP 26 of the UNFCCC. Thus, the presidents of the CBD and UNFCCC, China and the UK with Italy, could support cooperation and financing facilities to support countries that wish to promote spatial planning for NBS in their national policy frameworks and LT-LEDS.
